# Supplementary material for: Competing Energy Scales in Topological Superconducting Heterostructures
Source: Nano Lett. 2021 Apr 1;21(7):2758–65. doi: 10.1021/acs.nanolett.0c04648 (PMC8155198; doi:10.1021/acs.nanolett.0c04648)
Supplement: Supplementary file 1 — nl0c04648_si_001.pdf [file nl0c04648_si_001.pdf]

Supplementary Information for: Competing energy scales in topological superconducting heterostructures

Yunyi Zang,[1] Felix Küster,[1] Jibo Zhang,[1] Defa Liu,[1] Banabir Pal,[1]
Hakan Deniz,[1] Paolo Sessi,[1] Matthew J. Gilbert,[2] and Stuart S.P. Parkin[1]

[1] *Max Planck Institute of Microstructure Physics, Halle 06120, Germany*

[2] *University of Illinois at Urbana-Champaign,*
Department of Electrical and Computer Engineering, Urbana, IL 61820, USA

(Dated: March 10, 2021)

Abstract

SUPPLEMENTARY NOTE 1:
GROWTH PARAMETERS

We used a homemade ultra-high vacuum (base pressure $p < 1 \cdot 10^{-10}$ mbar) molecular beam epitaxy (MBE) system to grow all the heterostructures. Nb (99.999 % purity) was grown on top of Al_2O_3, a substrate routinely used to create high quality metal–ceramic interfaces [1]. The substrate temperature was kept at $T = 1000$ K during the growth. X-ray diffraction indicates that the Nb film deposited on $Al_2O_3(11\bar{2}0)$ grows along the (110) direction. Achieving this surface orientation is important since it promotes the growth of the successive layers along their (111) direction [2, 3]. This makes our Nb/Al_2O_3 system an ideal platform for the growth of both Pt as well as conventional topological insulators such as Bi_2Te_3 along their (111) surface direction [4]. Pt (99.999 % purity) was deposited on Nb at $T = 300$ K. To create Bi_2Te_3 thin films, Bi (99.999 % purity) and Te (99.9999 % purity) were co-evaporated while keeping the Nb substrate at $T = 570$ K. The crystalline quality of the films was monitored by in-situ reflective high-energy electron diffraction (RHEED) (see Supplementary Figure 1) and ex-situ transmission electron microscopy (TEM) (see Supplementary Figure 2).

SUPPLEMENTARY FIGURE 1:

REFLECTION HIGH-ENERGY ELECTRON DIFFRACTION DURING GROWTH

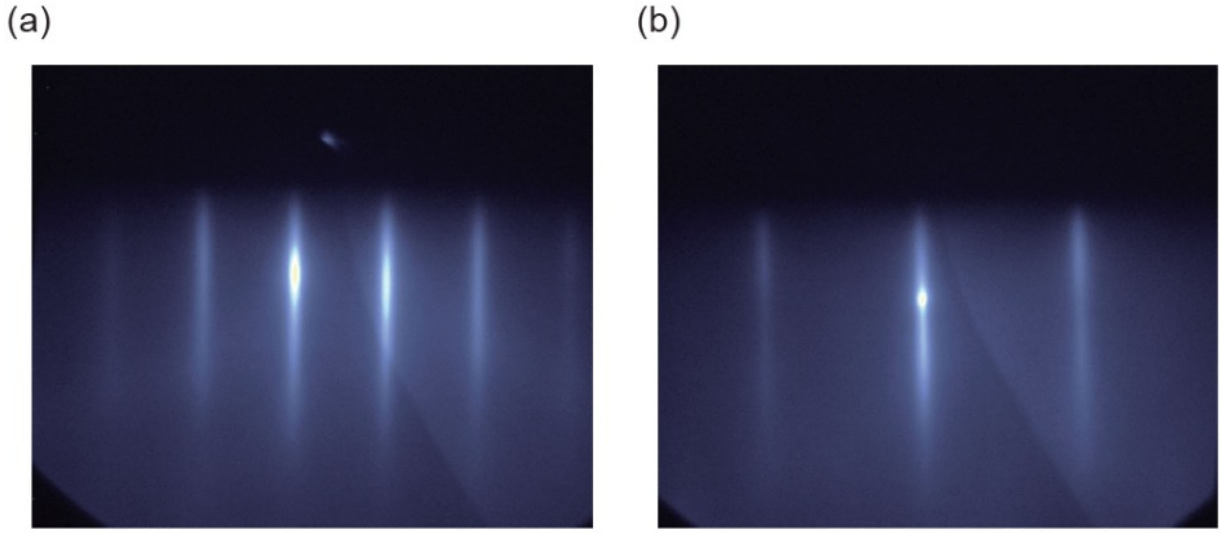

Figure S 1: Reflection high-energy electron diffraction (RHEED) patterns of epitaxial Bi_2Te_3 thin film grown on Nb(110). The sample consists of 5nm Bi_2Te_3 grown on 6.5nm Nb. (a) and (b) represent RHEED patterns from $[1\bar{1}0]$ and $[11\bar{2}]$ directions. Sharp and clear stripes demonstrate high quality of the material. The separation of the stripes in (a) and (b) give rise to a ratio of $1{:}\sqrt{3}$, suggesting the six fold symmetry. The estimated lattice constant is a = 4.33 Å, in agreement with the lattice constant of Bi_2Te_3.

SUPPLEMENTARY FIGURE 2:

TRANSMISSION ELECTRON MICROSCOPY CHARACTERIZATION

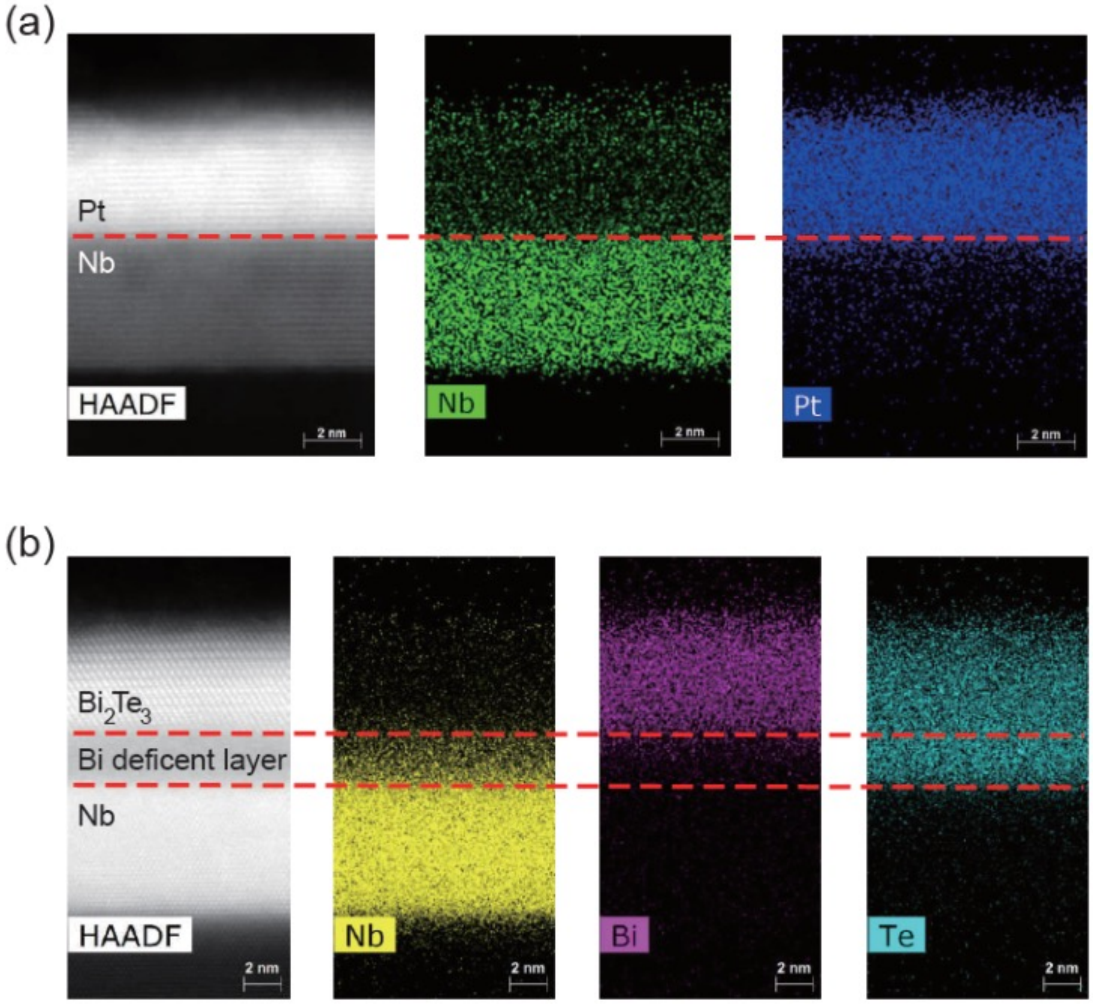

Figure S 2: Transmission electron microscopy (TEM) of epitaxial Pt and Bi_2Te_3 thin films grown on Nb(110). (a) and (b) report the results of the structural and chemical analysis of the Pt/Nb and Bi_2Te_3/Nb heterostructures, respectively. The cross-sectional images of the heterostructures [left panels in (a) and (b)], have been acquired by high-angle annular dark-field imaging (HAADF). The chemical analysis has been performed by energy-dispersive X-ray spectroscopy (EDX) within the TEM set-up. Both heterostructures show sharp interfaces, demonstrating the high quality of our samples. In the case of Bi_2Te_3/Nb, the EDX maps reveal a Bi deficient layer which is spontaneously forming during the growth.

SUPPLEMENTARY FIGURE 3:

SPATIAL MAPPING OF THE SUPERCONDUCTING GAP

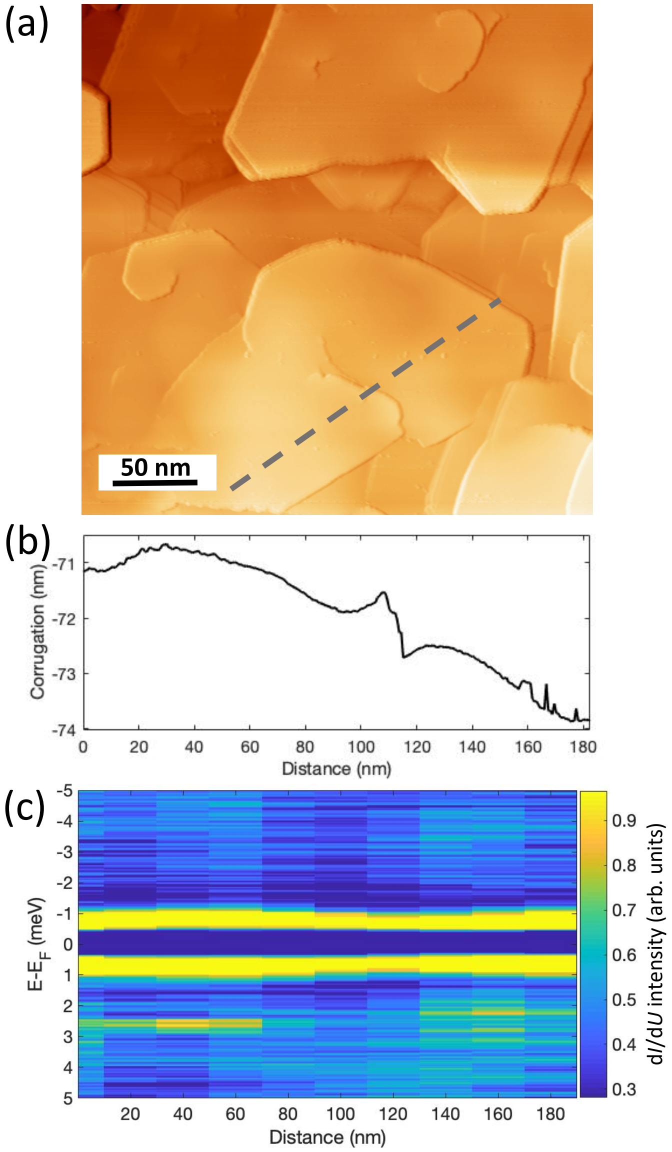

Figure S 3: Spatial mapping of the superconducting gap. (a) Topographic image of Bi_2Te_3 grown onto the Nb film; (b) line profile and (c) dI/dU spectroscopy of the superconducting gap taken along the grey dashed line in (a). Despite the presence of structural inhomogeneities such as step edges and screw dislocations, a narrow and homogeneous superconducting gap is present on the surface.

SUPPLEMENTARY FIGURE 4:

SPATIAL MAPPING OF THE SUPERCONDUCTING VORTICES

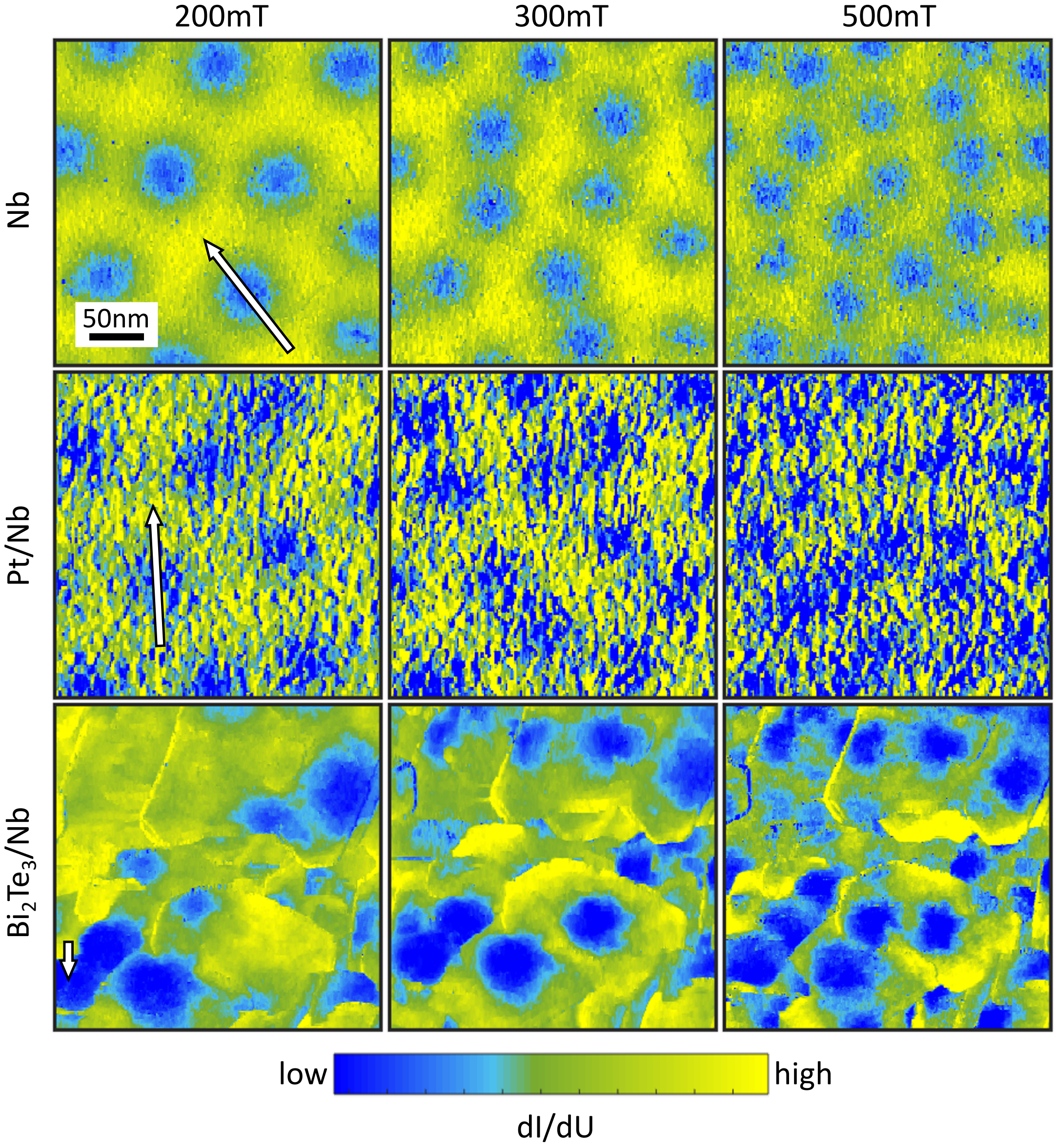

Figure S 4: Spatial mapping of the superconducting vortices. dI/dU maps acquired at progressively stronger magnetic fields applied perpendicular to the sample surface on (a) Nb, (b) Pt/Nb, and (c) Bi_2Te_3/Nb. All dI/dU maps have been acquired by setting the tunneling bias at the coherent peak energy. The white lines correspond to the positions where the spectroscopic maps of the superconducting vortexes reported in Figure 3 in the main text have been acquired.

SUPPLEMENTARY FIGURE 5:

SPECTROSCOPY ACROSS VORTICES

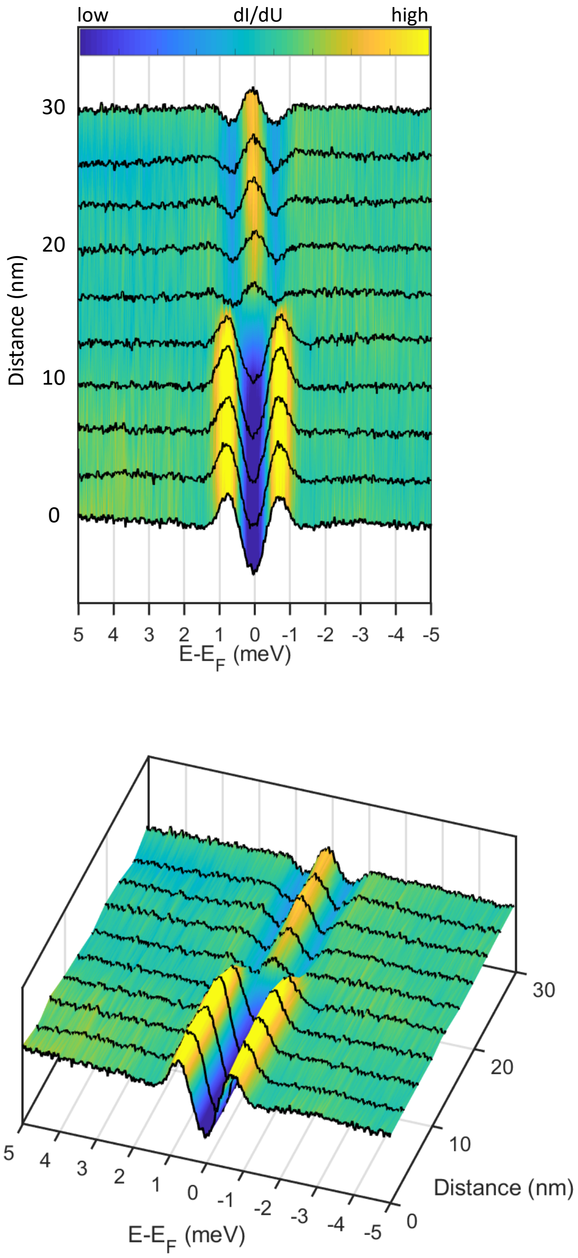

Figure S 5: Spectroscopy across vortices. dI/dU spectra (black lines) acquired across a superconducting vortex in Bi_2Te_3/Nb films (see white line Supplementary Figure 4. The spectra are shown under two different view angles to highlight the sharp transition from the superconducting gap to the zero-bias-conductance-peak. They have been used to generate the plot reported in Fig.3i in the main text.

SUPPLEMENTARY FIGURE 6:

SPATIAL MAPPING OF THE SUPERCONDUCTING VORTICES

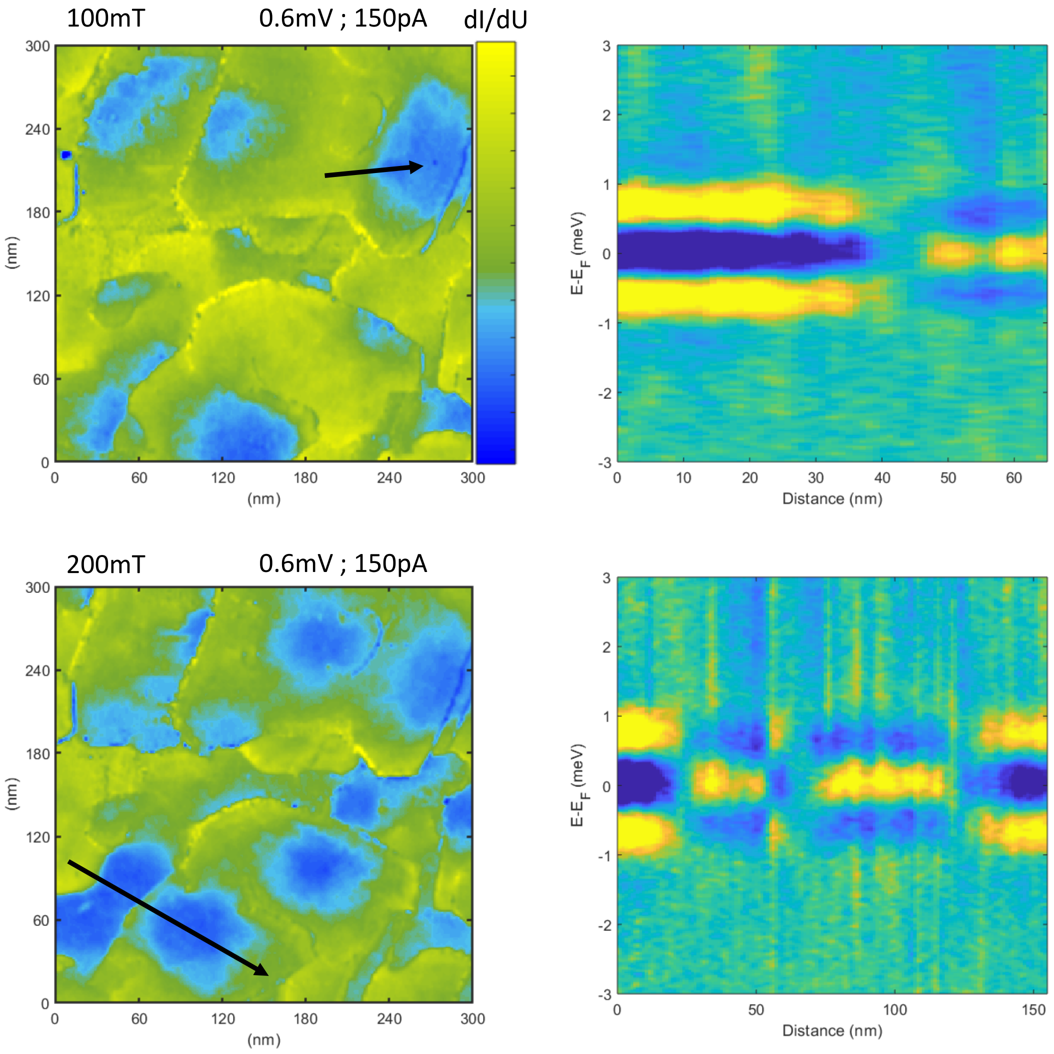

Figure S 6: Line-spectroscopy data acquired across different vortices. Line-spectroscopy data acquired across different vortices and at different magnetic fields confirm the existence of a zero-bias-peak emerging with a relatively sharp transition from the superconducting gap. Consistently with the data reported in Fig.3i, these additional data also confirm that the zero-bias-peak does not split right off the vortex center.

SUPPLEMENTARY NOTE 2:
MODELING TOPOLOGICAL SUPERCONDUCTING HETEROSTRUCTURES

To understand the physics of unmasked in the vortices measured in proximity-coupled s-wave superconductors - 3D time-reversal invariant topological insulators (STI) system, we begin with the Hamiltonian for the 3D topological insulator phase written in the real-space representation as

$$H_{TI} = \sum_{\vec{k}} c^\dagger_{\vec{k}} H_{TI}(\vec{k}) c_{\vec{k}} \tag{1}$$

$$= \sum_{\vec{k}} c^\dagger_{\vec{k}} \left[d_\alpha(\vec{k})\Gamma^\alpha + M(\vec{k})\Gamma^0 \right] (\vec{k}) c_{\vec{k}}. \tag{2}$$

In Eq. (2), $\alpha = 1, 2, 3$ to denote the appropriate gamma matrix, which we define here as $\Gamma^\alpha = \tau^x \otimes \sigma^\alpha$ and $\Gamma^0 = \tau^z \otimes \mathbb{I}_{2\times 2}$. In the definition of the (4×4) Gamma matrices, τ^α represents the orbital degree of freedom (A, B) and σ^α represents the spin degree of freedom $(\uparrow, \downarrow)$ corresponding to a complete basis that we define as $c_{\vec{k}} = \left[c_{\vec{k}A\uparrow}, c_{\vec{k}A\downarrow}, c_{\vec{k}B\uparrow}, c_{\vec{k}B\downarrow} \right]^T$. In order to be able to coherently interpret the measured results, we utilize both the lattice and continuum forms of Eq. (2) in which certain parameters within the Hamiltonian will acquire different definitions. In particular, in the continuum description, the momentum dependent term $d_\alpha(\tilde{\mathbf{p}})$ is defined to be

$$d_\alpha(\tilde{\mathbf{p}}) = \hbar v_F p_\alpha, \tag{3}$$

and the mass term, whose value will dictate the presence of absence of topological states in Eq. (2), is defined as

$$M(\tilde{\mathbf{p}}) = m_0 - \frac{1}{2} b p^2. \tag{4}$$

In the preceding definitions presented in Eq. (3) and Eq. (4), the parameters b, m_0 and v_F are all materials dependent parameters and may be adjusted to suit the specific properties of the system in question. In all subsequent calculations, we seek to have a qualitative understanding of the physics rather than more specific quantitative agreement, therefore, we set the combination of $\hbar v_F = 1$ without any loss in understanding. In a similar fashion to the situation for the continuum case, when we consider the definition of the lattice model that is associated with Eq. (2), we require a commensurate set of definitions for vectors d_α and M. In the lattice description of the model, we define these parameters to be

$$d_\alpha(\tilde{\mathbf{p}}) = \frac{\hbar v_F}{a_0} \sin\ (p_\alpha), \tag{5}$$

while

$$M(\tilde{\mathbf{p}}) = \frac{b}{a_0^2}\left[\cos\ (p_x a_0) + \cos\ (p_y a_0) + \cos(p_z a_0) - (1+m_0)\right]. \tag{6}$$

In Eq. (5) and Eq. (6), we require the addition of an additional parameter, a_0, that represents the lattice constant which we hereafter set to be unity without any loss of generality. The above model possesses time-reversal symmetry that is represented by $\mathbb{T} = \mathbb{I}_{2\times 2} i\sigma^y \mathbb{K}$ in which $\mathbb{K}$ represents the operation of complex conjugation. Regardless of referring to the lattice or continuum model, when the mass parameter, $m_0 \neq 0$ in either Eq. (4 or Eq. (6), then the Hamiltonian is that of a gapped insulator. With the use of the continuum model, we may, assuming that the system possesses translational invariance, calculate the resultant energy spectrum as

$$E = \pm\sqrt{p^2 + \left[m_0 - \frac{1}{2}bp^2\right]^2}. \tag{7}$$

In Eq. (7), we note that there are only two branches in the spectrum due to each of the branches being doubly degenerate. In keeping with the convention in dealing with 3D topological insulators such as Bi_2Se_3, as is the case here, it is common to set $b > 0$ thereby delineating the topological and trivial regimes to occur when $m_0 > 0$ and $m_0 < 0$ respectively. In the subsequent analysis, we will keep with the aforementioned parameter convention when describing topological and trivial phases.

With the essential model of the underlying topological insulator defined, we now require that the proximity-induced s-wave superconductivity and the magnetic effects be included in the model. Within the literature, there is growing consensus that Majorana states may be found in the center of vortex cores in proximity-coupled s-wave superconductor / 3D topological insulator heterostructures in the presence of a magnetic field, $\mathbf{B}$. Within the continuum model the addition of these terms may be done via the Bogoliubov-de Gennes (BdG) Hamiltonian that is composed of an 8×8 Nambu basis as

$$H_{SC/TI} = \frac{1}{2}\sum_{\tilde{\mathbf{p}}\Psi_{\tilde{\mathbf{p}}}} \begin{bmatrix} H_{TI}(\tilde{\mathbf{p}} - e\mathbf{A}) & \Delta \\ \Delta^\dagger & H_{TI}(\tilde{\mathbf{p}} - e\mathbf{A}) \end{bmatrix} \Psi_{\tilde{\mathbf{p}}}. \tag{8}$$

In Eq. (8), $\mathbf{A}$ is the vector potential and Δ is the superconducting pairing magnitude which, for s-wave superconductivity, may be written as $\Delta(\vec{r}) = \Delta_0(\vec{r})\mathbb{I} \otimes i\sigma^y$ where this term effectively pairs opposite spin quasiparticles in the doubled Hamiltonian in basis $\Psi_{\tilde{\mathbf{p}}} = \left[c_{\mathbf{p}}\ c_{-\mathbf{p}^\dagger}\right]$. Given the experimental structure, we assume that the superconductive pairing

is homogeneous in the $x - y$-plane in the absence of vortices and will vary in the direction perpendicular to the the heterostructure, $\Delta_0(z)$, as there is only superconductivity on one side of the topological insulator. Thus, the superconductivity should be strongest near the interface and decay as we move to the surface, where the measurements are taken. Considering previous work, we understand that in the presence of a magnetic field, the superconductor will proliferate vortices on the surface that may be accounted for in our theory by a winding of the superconducting order parameter, $\Delta_0(\tilde{\mathbf{r}}) = \Delta_0 e^{i\theta(\tilde{\mathbf{r}}}$, where $\theta(\tilde{\mathbf{r}})$ is the polar angle. Therefore, the desired result of the introduction of superconductivity on the surface of the topological insulator will be to open a gap in the energy spectrum of the metallic surface states upon which the proximity-induced vortices, in the presence of a uniform external magnetic field, will host the mid-gap Majorana states in the core.

Prior to the applying the theoretical constructs to the experimental results, we need to more clearly understand the superconducting component of the STI heterostructure as we intend to use it as the source of the vortices that may host the aforementioned Majorana fermions in the topological insulator. The physics of the magnetic flux within the superconductor is governed by the London equation,

$$\mathbf{B}(\tilde{\mathbf{r}}) - \lambda^2 \nabla^2 \mathbf{B}(\tilde{\mathbf{r}}) = \frac{\phi_0}{2} \delta(\tilde{\mathbf{r}}), \tag{9}$$

where $\mathbf{B}(\tilde{\mathbf{r}})$ is the magnetic field, ϕ_0 is the flux, and λ is the penetration depth. Assuming that we have a vortex positioned within our system at the origin, we may solve the London equation for the magnetic field

$$\mathbf{B}(\tilde{\mathbf{r}}) = \frac{\phi_0}{4\pi\lambda^2} K_0 \left(\frac{\tilde{\mathbf{r}}}{\lambda} \right), \tag{10}$$

where $K_n(x)$ are modified Bessel functions of the second kind, and the magnetic flux within a corresponding radius of r is given by

$$\phi(\tilde{\mathbf{r}}) = \left[\frac{1}{2} - \left(\frac{r}{2\lambda} \right) K_1 \left(\frac{\tilde{\mathbf{r}}}{\lambda} \right) \right]. \tag{11}$$

In both our continuum and numerical lattice models, we consider two different cases of the above defined penetration depth, λ, in our analysis: the "thin-flux" limit and the "thick-flux" limit[5]. Naturally, in a consideration of magnetic penetration in a superconductor, both $\lambda(z)$, $\Delta(z)$ acquire a position dependence that should be taken care of in a self-consistent fashion. In this work, we instead opt to explore the qualitative physics of the

STI heterostructure by ignoring the exact functional position dependence of the penetration depth and magnitude of the superconducting order parameter and instead using a presumed dependence for each quantity. Nonetheless, the two limits refer to distinct yet adiabatically connected regions of parameter space. Specifically, the "thin-flux" limit occurs when the topological insulator layer is sufficiently thin and the magnetic flux that penetrates into the bottom layer of the topological insulator does not have sufficient distance to spread before reaching the top surface. Within the parameters that we have defined for the STI system, the "thin-flux" limit occurs when $\lambda \approx a$. In the "thick-flux" limit, that occurs when $\lambda \gg a$, the penetrating flux is dispersed uniformly within the TI film prior to reaching the top surface.

In an effort to connect our theoretical framework to the experimental STI, we introduce a single π-flux tube into the STI and proceed by applying $k \cdot p$ perturbation theory. Within perturbation theory, we treat the momentum in the $\hat{z}$-direction, p_z as a perturbation within the basis of wavefunctions corresponding to the zero energy eigenstates, psi_0^1 and psi_0^2. As seen previously[5], the gapless Hamiltonian for the flux tube is $H_{flux} = p_z\sigma^x$ which corresponds to the insertion of a quantum spin Hall edge state into the STI. We desire to understand the evolution of the behavior of the zero energy eigenstates as we modify λ to increasingly large numbers compared to the lattice constant thereby efficiently traversing the "thin-flux" to "thick-flux" limits. As the penetration depth is increased, we expect the addition of a mass term that monotonically increases with increasing λ. Therefore, the flux tube Hamiltonian including the effects of λ induced masses m_x and m_y may be written as

$$H_{flux} = p_z\sigma^x + m_x(\lambda)\sigma^y + m_y(\lambda)\sigma^z. \tag{12}$$

The resultant energy spectrum for H_{flux} is $E_\pm\sqrt{p_z^2 + m_x^2 + m_y^2}$ corresponding to the expected result of a gapped spectrum at $p_z = 0$ in the presence of broken time-reversal symmetry breaking. Clearly the size of the gap that is induced by the presence of the flux, $E_{gap}^{ind} = \sqrt{m_x^2 + m_y^2}$ is governed by Eq. (11). In order to determine the ultimate size of the gap we E_{gap}^{ind}, we may apply perturbation theory. Nonetheless, in order to apply perturbation theory, we require a linearized version of Eq. (2 in the presence of a magnetic flux, here written where terms that are linear in momentum are kept

$$H_{linear} = m\tau_z \otimes \mathbb{I} + (p_x - eA_x)\tau_x \otimes \sigma_x + (p_y - eA_y)\tau_x \otimes \sigma_y + p_z\tau_x \otimes \sigma_z. \tag{13}$$

In Eq. (13), we have explicitly used the magnetic vector potential $\vec{A} = \frac{\phi\hbar}{r^2 e}(-y\hat{x}+x\hat{y})$ written in the symmetric gauge. Given the symmetries of STI system, particularly in the presence of vortices, we my now rewrite the linear Hamiltonian in Eq. (13) in polar coordinates as

$$H_{linear} = \begin{bmatrix} m & 0 & 0 & P_{-\theta} \\ 0 & m & P_\theta & 0 \\ 0 & P_{-\theta} & -m & 0 \\ P_\theta & 0 & 0 & -m. \end{bmatrix} \tag{14}$$

In Eq. (14), the two momenta components expressed, P_θ and $P_{-\theta}$, may be written as

$$P_\theta = \exp(i\theta)\left[\frac{\partial}{i\partial r} + \frac{\partial}{r\partial\theta} - \frac{i\phi}{r}\right] \tag{15}$$

and

$$P_{-\theta} = \exp(-i\theta)\left[\frac{\partial}{i\partial r} - \frac{\partial}{r\partial\theta} + \frac{i\phi}{r}\right] \tag{16}$$

Utilizing Eq. (14) in conjunction with first-order perturbation theory results in a corresponding first-order approximation for the induced gap resulting from the insertion of flux within the continuum model[6] as

$$E_{gap}^{ind} = \frac{\pi}{\lambda\alpha^2}\int_a^\infty K_1\left(\frac{r}{\lambda}\right)\exp\left(-2\int_a^r m dr'\right)dr. \tag{17}$$

To complete the picture of the physics of the STI with the continuum approach, we must now expand our Hamiltonian to be able to include the proximity-induced s-wave superconductivity within the simplified flux-line picture of Eq. (11) as

$$H_{flux}^{SC}(p_z) = \frac{1}{2}\begin{bmatrix} H_{flux}(p_z) & i\Delta\sigma^y \\ -i\Delta^*\sigma^y & -H_{flux}^*(-p_z). \end{bmatrix} \tag{18}$$

The energy spectrum of Eq. (18) consists of four non-degenerate bands,

$$E_{SC}^{thin} = \pm\sqrt{p_z^2 + \left(|\Delta| \pm E_{gap}^{ind}\right)^2}, \tag{19}$$

where the spectrum is gapped so long as $|\Delta| < |E_{gap}^{ind}|$ and represents the solution within the "thin-flux" regime.

However, the continuum model has limitations in that we cannot easily study the "thick-flux" limit due to the spatial dependence of the superconducting magnitude, Δ, as the flux spreads out in the topological film. Therefore, we must instead turn to a numerical

model based on the lattice representation of the STI in order to be capable of understanding the physics of the "thick-flux" regime and the interpolation between the "thin-flux" and "thick-flux" regimes. We begin the extension of the theoretical model within the lattice representation where we rewrite Eq. (2) in a slightly different manner in order to emphasize the on-site and lattice contributions in the position representation as

$$H_{TI} = \sum_{\tilde{\mathbf{r}}} c_r^\dagger H(m) c_r + \sum_{\tilde{\mathbf{r}},\delta} c_r^\dagger H(\delta) c_{r+\delta}, \tag{20}$$

with $H(m) = m_0 - 3b$ and $H(\delta) = \frac{b\Gamma_0 + iA\vec{\delta}\cdot\vec{\Gamma}}{2}$[5]. In Eq. (20), $\vec{r}$ denotes the lattice position, $\delta = [\pm a_0\hat{x}, \pm a_0\hat{y}, \pm a_0\hat{z}]$ describes nearest neighbor hopping between lattice sites, and $\vec{\Gamma} = \Gamma_1\hat{x} + \Gamma_2\hat{y} + \Gamma_3\hat{z}$. As we consider the interface between the topological insulator and a type-II s-wave superconductor, we recognize that there must be an even number of vortices present on the surface as a result of the periodic boundary conditions in our numerical solution[5, 7, 8]. Furthermore, we expect that the coupling between these disparate materials is sufficiently strong so that the superconducting Cooper pairs may tunnel from the superconductor to the topological insulator thereby imparting a superconducting paring potential within the inherently non-superconducting topological insulator. In order to account correctly for the superconductive pairing that is induced in the topological insulator near the interface between the two materials, we expand the Hamiltonian in Eq. (20) as

$$\begin{aligned} H_{BdG} = &\sum_{\tilde{\mathbf{r}}} \begin{pmatrix} c_{\tilde{\mathbf{r}}}^\dagger & c_{\tilde{\mathbf{r}}} \end{pmatrix} \begin{bmatrix} H(m) & \Delta_0 \mathbb{I}_{2\times2} i\sigma^y e^{i\phi(\tilde{\mathbf{r}})} \\ \Delta_0 \mathbb{I}_{2\times2} i\sigma^y e^{i\phi(\tilde{\mathbf{r}})} & -H^*(m) \end{bmatrix} \begin{bmatrix} c_{\tilde{\mathbf{r}}} \\ c_{\tilde{\mathbf{r}}}^\dagger \end{bmatrix} \\ + &\sum_{\tilde{\mathbf{r}},\delta} \begin{pmatrix} c_{\tilde{\mathbf{r}}}^\dagger & c_{\tilde{\mathbf{r}}} \end{pmatrix} \begin{bmatrix} H(\delta) e^{i\frac{e}{\hbar}\int_r^{r+\delta} \mathbf{A}(\mathbf{r})\cdot dl} & 0 \\ 0 & -H^*(\delta) e^{i\frac{e}{\hbar}\int_r^{r+\delta} \mathbf{A}(\mathbf{r})\cdot dl} \end{bmatrix} \begin{bmatrix} c_{\tilde{\mathbf{r+\delta}}} \\ c_{\tilde{\mathbf{r+\delta}}}^\dagger \end{bmatrix} \end{aligned} \tag{21}$$

.

In Eq. (21), $\mathbf{A}$ is the vector potential resulting from the presence of the magnetic field, λ represents the London penetration depth, ϕ_0 is the flux quantum and where the penetrating magnetic field in a superconductor satisfies the London equation.

Within the formulation of the Hamiltonian in Eq. (21) and the London equation in Eq. (9), the phase, $\phi(\tilde{\mathbf{r}})$ is a gauge field, however due to the presence of the proliferated vortices, the gauge is not a pure one. Therefore, we perform a gauge transformation to move the phase to the diagonal terms in the Hamiltonian. As the singular gauge transformation considers

each of the two vortices on a surface, then the gauge transformation is considered to be "bipartite"[7]. More specifically, the "bipartite" gauge transformation operates on both the particle and hole part of Eq. (21) and its main purpose here is to avoid an unclear multivalued definition for the line integral of the flux[5, 7], which must retain path independence up to an integer multiple of 2π. The full BdG Hamiltonian thus becomes

$$H_{BdG} = \sum_{\tilde{\mathbf{r}}} \left(c^{\dagger}_{\tilde{\mathbf{r}}} \; c_{\tilde{\mathbf{r}}} \right) \begin{bmatrix} H(m) & \Delta_0 \mathbb{I}_{2\times 2} i\sigma^y \\ -\Delta_0 \mathbb{I}_{2\times 2} i\sigma^y & -H^*(m) \end{bmatrix} \begin{bmatrix} c_{\tilde{\mathbf{r}}} \\ c^{\dagger}_{\tilde{\mathbf{r}}} \end{bmatrix}$$
$$+ \sum_{\tilde{\mathbf{r}},\delta} \left(c^{\dagger}_{\tilde{\mathbf{r}}} \; c_{\tilde{\mathbf{r}}} \right) \begin{bmatrix} H(\delta) e^{i\int_r^{r+\delta} \left[\nabla\phi_A(\mathbf{r}) - \frac{e}{\hbar c}\mathbf{A}(\mathbf{r}) \right]\cdot dl} & 0 \\ 0 & -H^*(\delta) e^{-i\int_r^{r+\delta} \left[\nabla\phi_A(\mathbf{r}) - \frac{e}{\hbar c}\mathbf{A}(\mathbf{r}) \right]\cdot dl} \end{bmatrix} \begin{bmatrix} c_{\tilde{\mathbf{r}+\delta}} \\ c^{\dagger}_{\tilde{\mathbf{r}+\delta}} \end{bmatrix} \quad (22)$$
.

With the Hamiltonian now fully defined, we proceed to examine the proximity-coupled STI heterostructures in relation to the experimental measurements of the vortices on the surface of thin-film systems. In Fig. **7**, we show the results of the numerical diagonalization of Eq. (22) on a 3D lattice of size $n_x = 28$ in the $\hat{x}$-direction, $n_y = 20$ in the $\hat{y}$-direction, and $\hat{z}$-direction each with a corresponding lattice constant of $a_0 = 1$. Periodic boundary conditions are applied to the $\hat{x}$ and $\hat{y}$-directions while the $\hat{z}$-direction has open boundary conditions. In keeping consistency between the experimental system and the theoretical model, we place the slab of topological insulator on top of an s-wave superconductor where we assume that the superconducting pairing potential decays as we move vertically away from the surface of the superconductor and further into the topological insulator in the $\hat{z}$-direction. Within the $x-y$-plane, we add two vortices corresponding to magnetic flux penetration from the superconductor, where the flux is tightly confined, to the topological insulator, where we assume the magnetic flux need not remain collimated. For all of the numerical results that we show here, we set $m_0 = 1.5$, $b = A = \hbar v_F = 1$ so as to clearly ensure that the resultant phase may be characterized as the strong topological insulator phase. Regarding the underlying superconductor, we set the initial pairing magnitude, $\Delta = 1$ and, we assume that the penetration depth, λ is a parameter that we may set and that it is dependent on the thickness of the topological insulator in the $\hat{z}$-direction.

With the system parameters defined, we now examine the numerical results in Fig. (**7**). Examination of Fig. (**7**), demonstrates the results of diagonalization of Eq. (22). The solution grid we employ corresponds to a numerical lattice of size $n_x = 28$ points in the

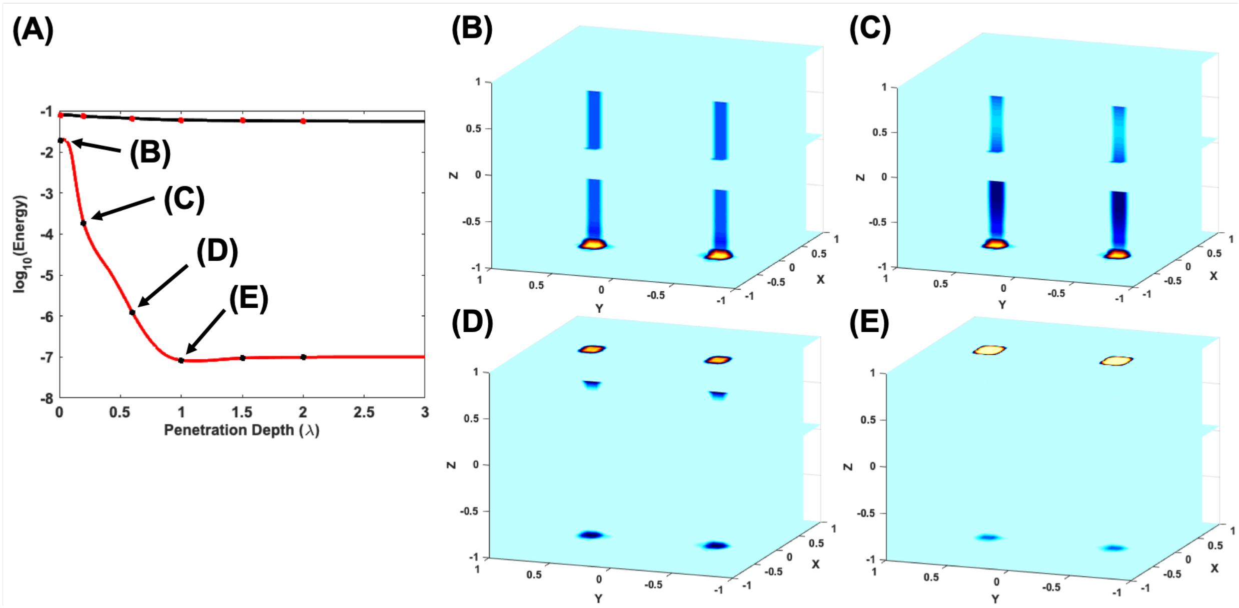

Figure S 7: Probability Distribution of Lowest Energy States in 3D: The probability density distributions corresponding to penetrations depths of (a) $\lambda = 0.01a$ (b) $\lambda = 0.1a$ (c) $\lambda = 0.5a$ and (d) $\lambda = 1.0a$ showing the evolution of the states from being delocalized along the magnetic flux tube to localized in the vortex cores on the surface as the penetration depth of the superconductivity is increased. (e) Plot of the energy for the lowest and second lowest energy modes in the 3D system for $\mu = 0.2$. We observe the formation of the topological phase in which the energy of the lowest lying state, the Majorana fermion localized at the vortex core, approaches zero with increasing λ while the trivial second lowest state remains constant in energy as the penetration depth is changed.

$\hat{x}$-direction, $n_y = 20$ points in the $\hat{y}$-direction and $n_z = 24$ points in the $\hat{z}$-direction with the lattice constant $a = 1$ for all of the simulations performed in this work. Furthermore, the superconducting order parameter decays away from the surface of the superconductor linearly dropping to zero within 6 layers of the surface of the superconducting interface. We have examined a range of superconducting magnitude decays as a function of position as we move away from the superconductor-topological insulator interface and find that the results presented in Fig. (**7**) remain largely unchanged.

In Fig. (**7**)(a), we plot the energies of the two lowest lying states in the STI heterostructure as the penetration depth, λ, varies in magnitude represented in units of the lattice constant, a. The range of λ considered sweeps the range of the "thin-flux" limit to the "thick-flux"

limit and, correspondingly, examine the changes in the discrepancy between the lowest and second lowest energies in the STI heterostructure. Clearly, as the penetration depth is increased we move from the "thin-flux" limit to the "thick-flux" limit, in which the Majorana modes are localized on the surfaces of the STI[9]. Fig. (**7**)(b) illustrates the wavefunction for the lowest lying state in the STI heterostructure within the "thin-flux" limit where the physics is dominated by the wormhole effect that allows Majorana states on the top surface be delocalized between the surfaces creating a gap that pushes the energy of the lowest lying state towards the second lowest lying energy state for small λ. As we increase λ we observe that the lowest lying energies in the system are exponentially decreasing as they approach the ideal, or zero energy. In the intermediate figures Fig. (**7**)(c) and (d) corresponding to the points of λ that interpolate between the "thin-flux" regime and the "thick-flux" regime, we observe that the weight of the wavefunction shifts towards the top and bottom surfaces indicating that the states are becoming more localized along the surfaces of at the top and bottom of the topological film. In Fig. (**7**)(e), we observe for $\lambda = 1$ that the modes are now localized on the surfaces with no weight of the wavefunction remaining in the bulk of the topological material.

[1] A. R. Wildes, J. Mayer, and K. Theis-Bröhl, Thin Solid Films **401**, 7 (2001).

[2] J. Kwo, M. Hong, and S. Nakahara, *Applied Physics Letters*, Applied Physics Letters **49**, 319 (1986).

[3] P. B. Welander, *Journal of Applied Physics*, Journal of Applied Physics **108**, 103508 (2010).

[4] C.-L. Song, Y.-L. Wang, Y.-P. Jiang, Y. Zhang, C.-Z. Chang, L. Wang, K. He, X. Chen, J.-F. Jia, Y. Wang, Z. Fang, X. Dai, X.-C. Xie, X.-L. Qi, S.-C. Zhang, Q.-K. Xue, and X. Ma, *Applied Physics Letters*, Applied Physics Letters **97**, 143118 (2010).

[5] C.-K. Chiu, M. J. Gilbert, and T. L. Hughes, Phys. Rev. B **84**, 144507 (2011).

[6] G. Rosenberg, H.-M. Guo, and M. Franz, Phys. Rev. B **82**, 041104 (2010).

[7] O. Vafek, A. Melikyan, M. Franz, and Z. Tešanović, Phys. Rev. B **63**, 134509 (2001).

[8] H.-H. Hung, P. Ghaemi, T. L. Hughes, and M. J. Gilbert, Phys. Rev. B **87**, 035401 (2013).

[9] L. Fu and C. L. Kane, Phys. Rev. Lett. **100**, 096407 (2008).
